# S07-2 Variability in physical activity levels in adolescent Gaelic football players across a competitive season

**DOI:** 10.1093/eurpub/ckac093.035

**Published:** 2022-08-29

**Authors:** Kevin Gavin

**Affiliations:** Sport Department, Athlone Institute of Technology, Athlone, Ireland

**Keywords:** health promotion, sports club, physical activity

## Abstract

**Background:**

Gaelic football is the most popular team-based sport among Irish adolescents, yet research examining the physical activity (PA) levels attained during this sport is limited. Moreover, no research has examined the PA levels of participants across a competitive season, limiting the data collection to one, often undisclosed time point. Therefore, the aim of this study was to objectively measure changes in PA levels attained by adolescents during Gaelic football participation across two time points in a season and determine if these changes had an effect on the overall daily PA levels.

**Methods:**

A total of 131 adolescents (67 male, 64 female; mean age 14.5 yrs.) were recruited from a convenience sample of three local clubs. Participants wore an activPAL3M accelerometer to determine total sitting/lying time, standing time, light intensity physical activity (LIPA) and moderate-to-vigorous physical activity (MVPA) during a seven-day measurement period, including Gaelic football participation. Physical activity measurement was completed at two separate time points, in line with the ?preseason? (round 1) and ?in-season? (round 2) periods of the Gaelic football calendar.

**Results:**

Males accumulated significantly less time in MVPA during games in round 2 when compared to round 1 (round 1 = 36%; round 2 = 30%; p > 0.05). In contrast, females accumulated significantly more time in MVPA during both practices (round 1 = 19%; round 2 = 26%; p > 0.05) and games (round 1 = 27%; round 2=35%; p > 0.05) during round 2. The average daily minutes spent in MVPA by males significantly decreased between the two time points (round 1 = 36.5±12.9min; round 2 = 30.8±13.5min; p > 0.05), while daily MVPA in females significantly increased (round 1 = 26.3±8.9min; round 2 = 30.6±11.6min; p > 0.05). No significant differences in daily MVPA levels were observed between the genders during round 2 (p > 0.05).

**Conclusion:**

The results demonstrate that the PA levels attained during Gaelic football participation change significantly across a competitive season. These changes may be a direct result of different coaching strategies implemented as the season progresses. The overall daily PA levels of the participants also changed significantly across the time points, resulting in males and females accumulating equal amounts of MVPA daily. This change in daily PA levels may be a consequence of the changes in PA levels attained during Gaelic football participation.

